# A Rare Variant in *ERF* (rs144812092) Predisposes to Prostate and Bladder Cancers in an Extended Pedigree

**DOI:** 10.3390/cancers13102399

**Published:** 2021-05-15

**Authors:** Lisa Anne Cannon-Albright, Craig Carl Teerlink, Jeff Stevens, Franklin W. Huang, Csilla Sipeky, Johanna Schleutker, Rolando Hernandez, Julio Facelli, Neeraj Agarwal, Donald L. Trump

**Affiliations:** 1 Genetic Epidemiology, Department of Internal Medicine, University of Utah School of Medicine, Salt Lake City, UT 84112, USA; Craig.teerlink@hsc.utah.edu (C.C.T.); jstevens@genetics.utah.edu (J.S.); 2 Huntsman Cancer Institute, University of Utah, Salt Lake City, UT 84108, USA; Neeraj.agarwal@hci.utah.edu; 3 George E. Wahlen Department of Veterans Affairs Medical Center, Salt Lake City, UT 84148, USA; 4 Division of Hematology/Oncology, Department of Medicine, Helen Diller Family Comprehensive Cancer Center, Bakar Computational Health Sciences Institute, Institute for Human Genetics, University of California, San Francisco, CA 94143, USA; Franklin.huang@ucsf.edu; 5 Institute of Biomedicine and FICAN West Cancer Centre, University of Turku, Turku University Hospital, 20521 Turku, Finland; Csilla.sipeky@utu.fi (C.S.); Johanna.schleutker@utu.fi (J.S.); 6 UCB Pharma, Data & Translational Sciences, 1420 Braine l’Alleud, Belgium; 7 Department of Medical Genetics, Genomics, Laboratory Division, Turku University Hospital, 20521 Turku, Finland; 8 Department of Biomedical Informatics, University of Utah School of Medicine, Salt Lake City, UT 84108, USA; rolando.hernandez@utah.edu (R.H.); Julio.facelli@utah.edu (J.F.); 9 Center for Clinical and Translational Science, University of Utah School of Medicine, Salt Lake City, UT 84112, USA; 10 Division of Oncology, Department of Internal Medicine, University of Utah School of Medicine, Salt Lake City, UT 84132, USA; 11 Inova Schar Cancer Institute, Inova Health System, 8081 Innovation Park Drive, Fairfax, VA 22031, USA; skip2dornoch1@gmail.com; 12 Department of Medicine and Cancer Center, University of Virginia, Charlottesville, VA 22903, USA

**Keywords:** bladder cancer, UPDB, high-risk pedigree, *ERF*, prostate cancer, predisposition

## Abstract

**Simple Summary:**

Here we applied a powerful predisposition candidate gene identification strategy to identify rare variants shared by two related bladder cancer cases who were members of pedigrees exhibiting a significant excess of bladder cancers. We sequenced the exomes of pairs of related bladder cancer cases belonging to high-risk bladder cancer pedigrees to identify rare, shared variants shared as candidates for predisposition. A rare, shared variant in *ERF* was also found to show significant association with bladder cancer risk in an independent population, was present in other prostate cancer-affected members in the pedigree, and showed evidence for altering the function of the associated protein. This evidence supports *ERF* (ETS2 Repressor Factor) as a bladder and prostate cancer predisposition gene.

**Abstract:**

Pairs of related bladder cancer cases who belong to pedigrees with an excess of bladder cancer were sequenced to identify rare, shared variants as candidate predisposition variants. Candidate variants were tested for association with bladder cancer risk. A validated variant was assayed for segregation to other related cancer cases, and the predicted protein structure of this variant was analyzed. This study of affected bladder cancer relative pairs from high-risk pedigrees identified 152 bladder cancer predisposition candidate variants. One variant in *ERF* (ETS Repressing Factor) was significantly associated with bladder cancer risk in an independent population, was observed to segregate with bladder and prostate cancer in relatives, and showed evidence for altering the function of the associated protein. This finding of a rare variant in *ERF* that is strongly associated with bladder and prostate cancer risk in an extended pedigree both validates *ERF* as a cancer predisposition gene and shows the continuing value of analyzing affected members of high-risk pedigrees to identify and validate rare cancer predisposition variants.

## 1. Introduction

Bladder cancer is not often recognized to cluster in families, and inherited variants are not thought to be a major risk factor, although an inherited contribution to predisposition has been suggested [1,2,3]. Study of high-risk pedigrees is recognized as a powerful method to identify disease predisposition genes [4,5,6]. This high-risk pedigree approach has been previously successful in Utah in the identification of predisposition genes and variants for a variety of cancers [7,8,9,10,11]. Here we applied a powerful and efficient predisposition candidate gene identification strategy to identify rare variants shared by two related bladder cancer cases who were members of pedigrees exhibiting a significant excess of bladder cancers. From a biorepository of germline DNAs representing extended high-risk cancer pedigrees for different cancer types we identified sampled bladder cancer cases, identified all related clusters of sampled bladder cancer cases (pedigrees), and identified the subset of those pedigrees which exhibited a significant excess of bladder cancer cases. We sequenced the exomes of related pairs of bladder cancer cases from these high-risk bladder cancer pedigrees to identify rare variants shared in the affected case pairs as candidates for predisposition. A rare, shared variant in *ERF* identified as a candidate was also found to show significant association with bladder cancer risk in an independent population, it was present in other prostate cancer-affected members in the pedigree in which it was identified, and the variant was predicted to alter the function of the associated protein. *ERF* (ETS2 Repressor Factor) is a protein coding gene that is a member of the E26 transcription factor family which may regulate other genes involved in cellular proliferation.

## 2. Materials and Methods

### 2.1. Utah Population Data Base

The Utah Population Data Base (UPDB) resource includes the genealogy of the Utah founders in the mid-19th century to their modern-day descendants. Approximately 3 million individuals in the UPDB are part of at least three generations of genealogy that descends from a Utah founder. These individuals with extensive genealogy were analyzed here [12]. The UPDB links individuals to various Utah registries including the Utah Cancer Registry (UCR). The UCR has recorded all independent, primary cancers diagnosed or treated in Utah since 1966, and became an NCI Surveillance, Epidemiology, and End-Results (SEER) registry in 1973. Cancers are coded with International Classification of Disease (ICD) for Oncology. In the data analyzed here there are 148,885 individuals with at least one UCR record who have extended genealogy data; 5971 of these individuals have a diagnosis of bladder cancer.

### 2.2. Bladder Cancer Cases

A decades old biorepository was accessed to obtain the germline DNA samples analyzed here. This biorepository consists of DNA samples from ~36,000 members of Utah high-risk cancer pedigrees studied over many decades; many different cancer types were studied, and members of these pedigrees with cancers of any site were sampled when available. The DNA samples for 189 individuals who have linked genealogy data and a confirmed diagnosis of bladder cancer recorded in the UCR were identified; 79 of these bladder cancer cases also had a UCR confirmed diagnosis of prostate cancer. These individuals with both bladder and prostate cancer diagnoses were primarily ascertained for their membership in a high-risk prostate cancer pedigree and therefore are overrepresented in our ascertainment of sampled bladder cancer cases. All genetic relationships among the 189 sampled individuals with bladder cancer were analyzed to identify 103 independent descending pedigrees containing at least 2, and up to 11, of the sampled, related bladder cancer cases. By comparing the observed number of bladder cancer cases among the descendants in each of these pedigrees to the expected number (using bladder cancer rates in the UPDB population analyzed), 9 pedigrees that included a sampled pair of bladder-cancer-affected cousins and exhibited a significant excess of bladder cancer cases (high-risk pedigrees) were identified for analysis.

### 2.3. High-Risk Bladder Cancer Pedigrees

The sampled bladder cancer pedigrees at high-risk for bladder cancer were identified as follows. All ~3 million individuals in the UPDB with extended genealogy data as described above were assigned to a sex-, 5-year birthyear range-, and birth state- (Utah or not) cohort. The cohort-specific rate of bladder cancer was estimated for each cohort as the number of bladder cancer cases with genealogy data in the cohort divided by the total number of UPDB individuals with genealogy data in the cohort. The observed number of bladder cancer cases in the pedigree was counted; the expected number of bladder cancer cases in the pedigree was estimated by summing the cohort-specific rates of bladder cancer for all descendants in the pedigree. A statistical excess (*p* < 0.05) of the number of bladder cancer cases observed divided by the number of cases expected among the descendants was used to classify the pedigree as high-risk.

### 2.4. Whole Exome Sequencing

Whole exome sequencing (WES) was performed on the bladder cancer case cousin pairs from each of the nine high-risk pedigrees at the University of Utah Sequencing Facility. DNA libraries were prepared from 1.5 micrograms of DNA using the Agilent SureSelect Human All Exon V6+UTR capture kit. Samples were run on the Illumina HiSeq (San Diego, CA, USA) 2000 instrument. Reads were mapped to the human genome GRCh37 reference using BWA-mem for alignment and variants were called using Genome Analysis Toolkit version 3.6.0.1 (GATK) software (Cambridge, MA, USA) following Broad Institute Best Practices Guidelines. Exome capture resulted in an average of 87% of target bases being covered by greater than 10× coverage across the exome with an average depth of 90x. Variants were annotated with Annovar, which contains predicted pathogenicity scores from multiple in-silico functional prediction algorithms. Rare coding variants were selected with a cutoff frequency of ≤0.005. Each cousin pair was assessed individually for concordant rare variants.

### 2.5. Case-Control Association Analysis

Each of the rare candidate variants identified as shared in the bladder-cancer-affected cousins in at least one high-risk bladder cancer pedigree were considered independently for association with bladder cancer risk if there were variant data available in a set of 2294 bladder cancer cases and 22,940 ancestrally matched controls selected from UKBiobank (Stockport, UK). UKBiobank contains 488,377 total subjects genotyped on the Illumina OmniExpress high density SNP array [13]. The available genetic markers were reduced to a set of ~27 K independent markers, excluding several regions known to adversely affect principal component (PC) analysis [14]. PC eigenvectors for all 488,377 subjects were generated with FLASHPCA2 software [14]. Controls were selected from among 191,466 self-reported Caucasian subjects over age 70 years with no cancer diagnosis. Ten control subjects were selected for each bladder cancer case, selected from their nearest neighbors based on Euclidean distances of the first two PCs. Cases and ancestrally matched controls were imputed to ~40 M variants using Haplotype Reference Consortium’s (HRC) 67K background genomes [15]. Pre-imputation quality control (QC) was performed with PLINK software [16]. Subject QC required sample genotyping >98% and retained all subjects. QC of genetic markers began with 784,256 observed SNP genotypes. A total of 353,578 markers were removed by filtering for genotyping call rate <98%, HWE *p* < 1 × 10^−5^, MAF < 0.005, duplicated position in the HRC’s reference genome, or site not included in the HRC’s reference genome. The remaining QC-passing SNPs were converted to human genome B37 forward strand orientation with GenotypeHarmonizer software (Groningen, Netherlands) [17] and served as the basis for imputation. Imputation was performed with EAGLE v2.3 software for phasing [18] and MINIMAC3 software for imputation [19] with default settings on the HRC’s University of Michigan imputation server. The *ERF* variant was also considered for association with prostate cancer risk in a set of 5,129 Finnish prostate cancer cases and 3,506 cancer free controls; genotype data were imputed genomes of the iCOGS and OncoArray studies.

### 2.6. Protein Prediction Modeling

Following our previous approaches to demonstrate the usefulness of protein prediction methods to elucidate pathogenicity [10,20,21,22,23], the canonical/reference sequence for the *ERF* protein was retrieved from UniProt (Uniprot ID: P50548) [24]. The variant sequence was manually modified and the two resulting sequences were submitted to the Phyre2 server [25] on intensive mode for structure prediction. Two protein structures corresponding to the wild type and variant sequences were computed.

## 3. Results

The nine high-risk pedigrees selected for analysis each included a pair of bladder cancer-affected cousins (first to third-cousins); eight pedigrees had two sampled cases each and one pedigree had three sampled cases; one individual was in two independent pedigrees through different ancestors (total sequenced bladder cancer cases = 18). Exome sequencing of the 18 bladder-cancer affected individuals in the 9 extended pedigrees identified a total of 14,283 exonic variants in 7545 genes at MAF < 0.005 in EXAC. Of these, 6738 were non-synonymous, frameshift indel, stopgain or splice variants; 152 of these rare variants were concordant between at least one sequenced pair of bladder cancer-affected cousins. These 152 candidate bladder cancer predisposition variants are listed in Table S1.

Patients with Lynch syndrome carrying an *MSH2* variant are at increased risk of urinary tract cancer including bladder cancer [26]. No genetic screening results for any of the bladder cancer cases studied here were available. However, after sequencing, it was determined that the high-risk pedigree that included three bladder-cancer affected cousins had been previously studied as a high-risk colon cancer pedigree segregating a known PV in *MSH2*; two of the three cases shared the known *MSH2* PV segregating in the pedigree. 

Eighty-six of the 152 candidate variants had imputed data available and were tested for association with bladder cancer risk in the 2,294 bladder cancer cases and 22,940 controls from UKBiobank. Only 2 of the variants independently showed significant association with bladder cancer: *c19orf40* (rs36017455, OR = 2.33, *p* = 0.009) and *ERF* (rs144812092, OR = 3.64, *p* = 0.04). The *c19orf40* variant was observed in the 2 bladder-cancer cousin cases in which an *MSH2* PV was also observed, and was not pursued here. In the association study of Finnish *prostate cancer* cases and controls the *ERF* variant was observed in three cases and three controls (OR = 0.68, 94% CI 0.14, 3.39, *p* = 0.641). Only five prostate cancer cases had a family history, and none of these carried the variant.

**Figure 1:**
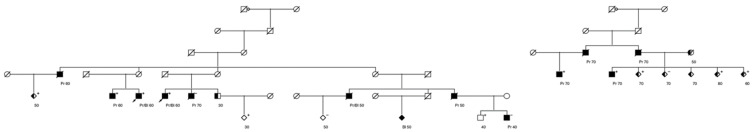
Pedigree segregating rare *ERF* variant. The male founder is shown with two marriages, indicated with an arrow on the marriage line. Sampled cancer cases and relatives are shown; assayed variant carriers are shown with “+”, the original sequenced bladder-cancer-affected cousin probands are indicated with an arrow. Prostate cancer cases are fully shaded, individuals with cancers of other sites are half-shaded; case details are censored to protect confidentiality.

The *ERF* variant rs144812092 was originally observed in a pair of bladder-cancer-affected first cousins. Each of these bladder cancer cases had also been diagnosed with prostate cancer decades before their bladder cancer diagnosis, which occurred in their late 70s and late 80s, respectively. The histology of the bladder cancers in the affected cousin pair were transitional cell carcinoma and papillary transitional cell carcinoma, respectively. The bladder cancer-affected cousin carriers were members of a previously sampled high-risk prostate cancer pedigree, shown in Figure 1. The pedigree is founded by a single male with two marriages. Additional members of the pedigree who had been previously sampled were assayed for the *ERF* variant (ThermoFisher (Waltham, MA, USA) assay: C__25967527_10) to test for segregation of the variant with cancer. Many additional carriers of the variant were identified, including seven additional carriers diagnosed with prostate cancer, and variant carriers diagnosed with both male and female breast cancer, lung cancer, leukemia, and lymphoma. As expected, the variant was not observed in all prostate cancer cases. This includes a prostate cancer case diagnosed in their late 40s who is a member of a branch in which variant carriers were observed. While this is surprising, there are many explanations for this observation, including the presence of additional predisposition variants in cases, mispaternity, or misdiagnosis. The male founder of the pedigree (with two spouses, both shown) was born in the early 1800s in Scotland and has >3500 descendants in the current UPDB (not all shown). Cancers observed in statistical excess among all descendants based on comparisons with cancer rates in the UPDB include: endometrial (RR = 2.4, *p* = 0.01) and prostate (RR = 1.47, *p* = 0.03); a borderline excess of bladder cancer cases (*n* = 7) was observed (RR = 1.79, RR = *p* = 0.10) in the pedigree. None of the other bladder cancer cases in the pedigree had samples available for assay.

**Figure 2:**
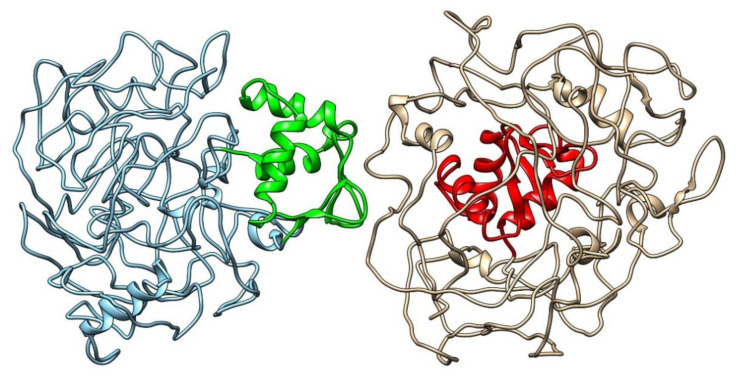
Two ERF wild type and variant structures (wildtype: blue; variant: tan) were compared side-by-side in UCSF Chimera. The DNA binding regions of the proteins are highlighted (wildtype: green; variant: red).

**Figure 3:**
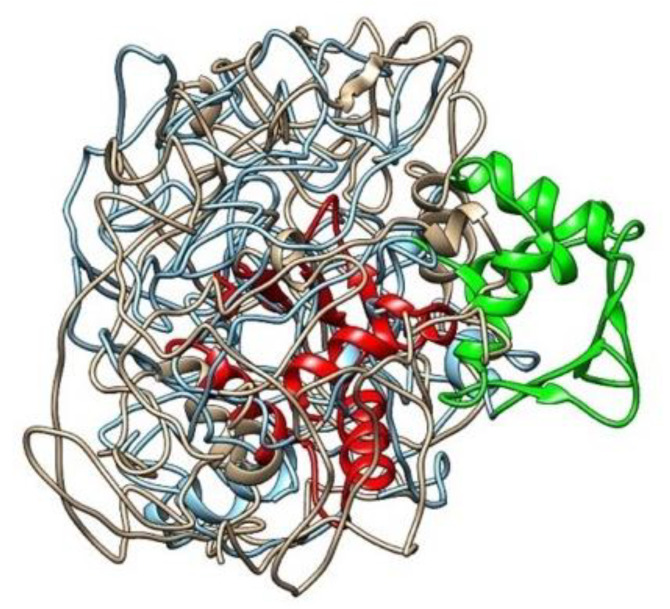
ERF structures were superimposed in UCSF Chimera and were found to be structurally dissimilar (RMSD across all pairs: 29.567 Å).

**Figure 4:**
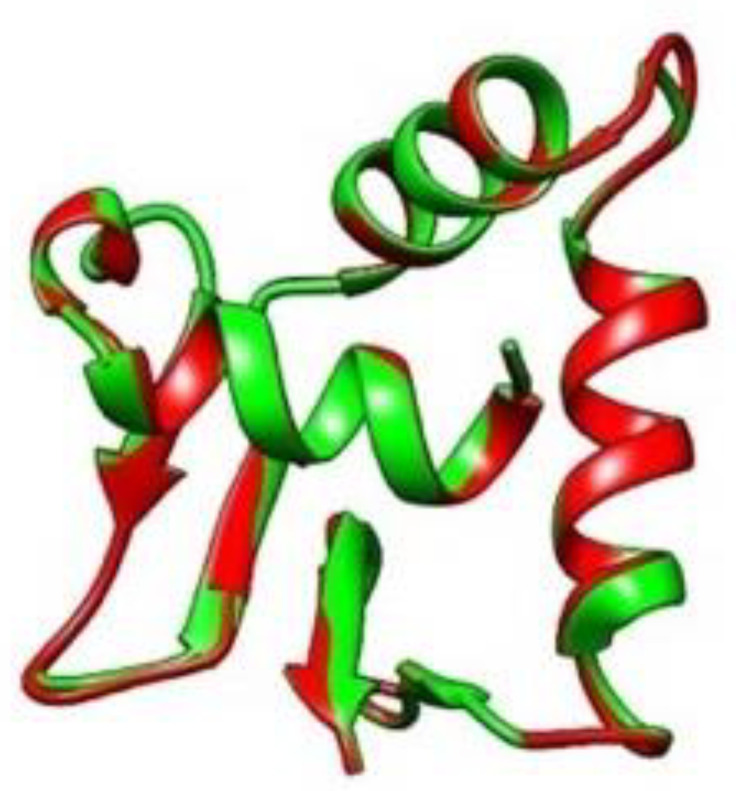
DNA binding domains of the wildtype and variant structures (green: wildtype; red: variant) were superimposed in UCSF Chimera and were found to be nearly identical (RMSD across all pairs: 0.058 Å).

In Figure 2, the blue image on the left corresponds to the wild type ERF isoform 1 protein, with the DNA binding region (residues 27–107) highlighted in green; the tan image on the right is the protein structure predicted for the variant considered here, with the single amino acid substitution Pro349Leu, where the DNA binding region is highlighted in red. This comparison shows a stark contrast in the placement of the DNA binding region (residues 27–107). While the binding region appears on the surface of the protein in the wild type, it is apparent that it moves inside the structure upon mutation, which could indicate a loss-of-function for the variant. The predicted structures show that the wild type DNA binding region is exposed to the solvent away from the rest of the intrinsically disordered regions, whereas in the variant, a contraction of the region into the core of the structure is observed. This suggests the variant sequence could cause the DNA binding region, necessary for transcription repression at the ETS2 promoter [24], to be disabled. This could be an indication of a loss-of-function variant which could contribute to pathogenesis. The connection to ETS2 is important because ETS2 is a transcription factor and protooncogene involved in development, apoptosis, and regulation of telomerase [27]. Figure 3 shows the two proteins superimposed; their structural dissimilarity was confirmed (RMSD across all pairs: 29.567 angstroms). Figure 4 shows the DNA binding regions of the two proteins superimposed; they were found to be nearly identically folded (RMSD across all pairs: 0.058 Å).

## 4. Discussion

Sequence analysis of a set of bladder cancer-affected cousin pairs who belonged to pedigrees with a significant excess of bladder cancer was performed to allow identification of rare, shared candidate bladder cancer predisposition variants. Analysis of available data from an independent population for the resulting set of candidate variants identified a variant in *ERF* (rs144812092) that was significantly associated with bladder cancer risk. This variant was also found to be present in multiple cancer-affected relatives of the original bladder-cancer-affected cousin pair, who were members of an extended high-risk prostate cancer pedigree. Protein prediction modeling of the variant suggested biologically meaningful effects to the protein. These results suggest the ERF variant (rs144812092) predisposes to bladder, prostate, and perhaps additional cancers observed.

*ERF* aliases include ETS domain-containing transcription factor EFR, and ETS2 Repressor Factor. ETS2 is a transcription factor and protooncogene involved in development, apoptosis, and regulation of telomerase; *ERF* acts as tumor suppressor by binding the protooncogene ETS2 promoter. *ERF* has been reported to be downregulated in prostate cancer. *ERF* rs144812092 (Chr19:42249066; GRCh38.p12) is a rare missense variant. Frequency estimates range from 0.00054 (64/117668, ExAC) to 0.00066 (162/246432, GnomAD_exome); 2 of 12 algorithms predict the variant as damaging; the GERP score = 2.42, and the variant has only been reported in ClinVar as benign in relation to craniosynostosis. These results may appear to contradict the pathogenic findings reported here; however, it has been recognized that the GERP score is not always a good indicator of pathogenicity [28], and that pathogenicity-predicting algorithms are highly influenced by the change of amino acid electrostatic properties upon substitution. For this mutation, Pro349Leu, both amino acids are non-polar and the structure in the vicinity of the mutation (Figure 4) does not change upon substitution. This may indicate that the pathogenicity can be attributed to steric effects (which are not considered in pathogenicity-prediction software) that render the binding domain to move inside the protein (Figure 2 and Figure 3) with the consequent loss of function due to inability to bind to DNA.

*ERF* has been identified as a prostate cancer tumor-suppressor gene in a study of localized primary prostate tumors from 102 African-Americans [29] in which recurrent loss-of-function somatic mutations in *ERF* were observed in 5% of cases. A germline analysis of *ERF* identified a different rare germline missense variant (S295I) in one high-risk prostate cancer patient in this cohort [29]. In existing prostate cancer cohorts *ERF* deletions were seen in 3% of primary prostate cancers and deletions of *ERF* were seen in 3–5% of lethal castration-resistant prostate cancers [30,31]. It was also reported that knockdown of *ERF* conferred increased anchorage-independent growth and generated a gene expression signature associated with oncogenic ETS activation and androgen signaling. Additionally, Bose [32] showed that recurrent point mutations and focal deletions of *ERF* cause decreased protein stability, and most occur in tumors without ERG upregulation; they argue that the oncogenicity of ERG is mediated, in part, by competition with *ERF*, and that overexpression of *ERF* blocks ERG-dependent tumor growth, and loss of *ERF* rescues TMPRSS2-ERG-positive prostate cancer cells from ERG dependency.

Limitations of this study include potential censoring, which could include individuals in the pedigree whose genealogy was not available or not linked, or individuals whose cancer was diagnosed outside Utah or before 1973. Utah’s founders were primarily of Northern European ancestry [33], so the candidate predisposition variants identified may not effectively or fully represent other populations. As noted, most of the sampled bladder cancer cases analyzed here also had a diagnosis of an independent primary prostate cancer based on their ascertainment and sampling as part of a prostate cancer high-risk pedigree study. Due to the low frequency of this variant (0.0005), association with prostate cancer risk will be difficult to show, as exhibited in the uninformative association analysis of the variant with prostate cancer in Finnish cases. Strengths of the study include the SEER quality cancer data, and the lack of ascertainment or recall bias for genealogy and cancer diagnosis data. The unique UPDB resource allows both identification and study of distant relationships, as well as validation of the high-risk nature of pedigrees.

## 5. Conclusions

In combination with previous work suggesting *ERF* as a prostate cancer gene, these observations additionally confirm the role of this rare *ERF* variant in familial prostate cancer. The observation of variant carriers exhibiting various cancers of other sites suggests a potential role in predisposition to more than just bladder and prostate cancers, but further studies are warranted. This study exemplifies the power and efficiency of the high-risk pedigree approach used to identify rare predisposition variants in high-risk cancer pedigrees as well as the use of powerful structural bioinformatics methods to provide mechanistic insights on pathogenesis.

## Data Availability

The data presented in this study are available on request from the corresponding author. The data are not publicly available due to data access requirements of UPDB.

## Supplementary Materials

The following are available online at https://www.mdpi.com/article/10.3390/cancers13102399/s1, Table S1. 152 rare candidate bladder cancer predisposition variants concordant between at least one sequenced pair of bladder cancer-affected cousins.
