# Supplementary material for: A Rare Variant in ERF (rs144812092) Predisposes to Prostate and Bladder Cancers in an Extended Pedigree
Source: Cancers (Basel). 2021 May 15;13(10):2399. doi: 10.3390/cancers13102399 (PMC8156789; doi:10.3390/cancers13102399)
Supplement: Supplementary file 1 [file cancers-13-02399-s001.zip › cancers-1175267-supplementary.pdf]

A Rare Variant in *ERF* (rs144812092) Predisposes to Prostate and Bladder Cancers in an Extended Pedigree

Lisa Anne Cannon-Albright, Craig Carl Teerlink, Jeff Stevens, Franklin Huang, Csilla Sipeky, Johanna Schleutker, Rolando Hernandez, Julio Facelli, Neeraj Agarwal and Donald L. Trump

Table S1. 152 rare candidate bladder cancer predisposition variants concordant between at least one sequenced pair of bladder cancer-affected cousins.

Chr	Start	End	Ref	Alt	Gene_refGene	ExonicFunc_refGene	ExAC_ALL
1	1133010	1133010	A	G	TTLL10	nonsynonymous SNV	1.00E-04
1	1391656	1391656	G	A	ATAD3C	nonsynonymous SNV	0.004
1	3413835	3413835	C	A	MEGF6	nonsynonymous SNV	8.00E-04
1	43316723	43316723	T	C	ZNF691	nonsynonymous SNV	.
1	52867839	52867839	C	G	ORC1	nonsynonymous SNV	0.0041
1	65124401	65124401	C	A	CACHD1	nonsynonymous SNV	.
1	1.86E+08	1.86E+08	C	T	TPR	nonsynonymous SNV	0.0036
1	2.03E+08	2.03E+08	C	T	CHIT1	nonsynonymous SNV	3.20E-03
1	2.04E+08	2.04E+08	C	T	LAX1	nonsynonymous SNV	0.0028
1	2.04E+08	2.04E+08	C	A	PIK3C2B	nonsynonymous SNV	.
1	2.34E+08	2.34E+08	A	C	KIAA1804	nonsynonymous SNV	0.0024
2	24302345	24302345	A	G	TP53I3	nonsynonymous SNV	0.00001647
2	33764243	33764243	C	A	RASGRP3	nonsynonymous SNV	1.11E-05
2	39025499	39025499	G	A	DHX57	nonsynonymous SNV	0.0042
2	47709944	47709944	C	-	MSH2	frameshift deletion	.
2	1.39E+08	1.39E+08	G	A	SPOPL	nonsynonymous SNV	0.0048
2	1.93E+08	1.93E+08	T	C	SDPR	nonsynonymous SNV	0.0013
2	2.28E+08	2.28E+08	A	G	MFF	nonsynonymous SNV	0.0001
3	405040	405040	C	T	CHL1	nonsynonymous SNV	3.31E-05
3	48802779	48802779	G	A	PRKAR2A	nonsynonymous SNV	0.00001988
3	1.97E+08	1.97E+08	G	A	PIGZ	nonsynonymous SNV	4.00E-04
4	515738	515738	C	T	PIGG	nonsynonymous SNV	0.0000661
4	946221	946221	G	A	TMEM175	nonsynonymous SNV	0.0022
4	1389003	1389003	G	C	CRIPAK	nonsynonymous SNV	0.0000688
4	15004992	15004992	C	T	CPEB2	nonsynonymous SNV	3.20E-03
5	35779248	35779248	A	G	SPEF2	nonsynonymous SNV	0.0021
5	38481703	38481703	G	T	LIFR	nonsynonymous SNV	0.0018
5	52403014	52403014	G	A	MOCS2	nonsynonymous SNV	0.0005
5	54558723	54558723	G	A	DHX29	nonsynonymous SNV	.
5	90059182	90059182	G	T	ADGRV1	nonsynonymous SNV	1.00E-03
5	1.14E+08	1.14E+08	T	C	TRIM36	nonsynonymous SNV	0.0005
5	1.4E+08	1.4E+08	G	C	PCDHA10	nonsynonymous SNV	0.0006
5	1.4E+08	1.4E+08	A	G	PCDHB3	nonsynonymous SNV	.
5	1.81E+08	1.81E+08	T	C	TRIM41	nonsynonymous SNV	.
6	41029294	41029294	C	A	APOBEC2	nonsynonymous SNV	0.005
6	42231009	42231009	G	A	TRERF1	nonsynonymous SNV	0.00002782
6	42905566	42905566	C	A	CNPY3	nonsynonymous SNV	0.000008241
6	65301693	65301693	C	T	EYS	nonsynonymous SNV	.
6	1.14E+08	1.14E+08	C	T	MARCKS	nonsynonymous SNV	0.0006
6	1.14E+08	1.14E+08	T	C	HS3ST5	nonsynonymous SNV	0.0003
6	1.19E+08	1.19E+08	C	T	MCM9	nonsynonymous SNV	0.00006528

6	1.68E+08	1.68E+08	A	G	HGC6.3	nonsynonymous SNV	1.30E-03
7	73183979	73183979	G	A	CLDN3	nonsynonymous SNV	0.0045
7	77166921	77166921	C	T	PTPN12	nonsynonymous SNV	0.00002317
7	81601108	81601108	C	T	CACNA2D1	nonsynonymous SNV	0.0027
7	1.4E+08	1.4E+08	G	A	TBXAS1	nonsynonymous SNV	0.0019
8	1.44E+08	1.44E+08	A	C	THEM6	nonsynonymous SNV	0.00007718
9	6553423	6553423	C	G	GLDC	nonsynonymous SNV	.
9	17462985	17462985	A	C	CNTLN	nonsynonymous SNV	0.0049
9	1.07E+08	1.07E+08	G	T	SMC2	nonsynonymous SNV	3.20E-03
9	1.13E+08	1.13E+08	C	T	AKAP2,PALM2-AKAP2	nonsynonymous SNV	2.00E-04
9	1.3E+08	1.3E+08	C	G	ZBTB43	nonsynonymous SNV	1.10E-03
9	1.41E+08	1.41E+08	G	C	ARRDC1	nonsynonymous SNV	0.001
10	46969292	46969292	G	A	SYT15	nonsynonymous SNV	0.0001
10	95993887	95993887	A	G	PLCE1	nonsynonymous SNV	9.00E-04
10	1.01E+08	1.01E+08	A	C	SLC25A28	nonsynonymous SNV	.
10	1.05E+08	1.05E+08	C	T	CALHM2	nonsynonymous SNV	0.0036
10	1.18E+08	1.18E+08	C	A	PNLIPRP3	nonsynonymous SNV	0.0026
10	1.3E+08	1.3E+08	G	A	MKI67	nonsynonymous SNV	0.0049
11	292060	292060	G	A	ATHL1	nonsynonymous SNV	0.001
11	640112	640112	A	G	DRD4	nonsynonymous SNV	0
11	640174	640174	A	G	DRD4	nonsynonymous SNV	8.00E-04
11	1651182	1651182	T	C	KRTAP5-5	nonsynonymous SNV	0.001
11	9068915	9068915	C	T	SCUBE2	nonsynonymous SNV	0.0015
11	13031880	13031880	A	G	RASSF10	nonsynonymous SNV	0.0009
11	1.03E+08	1.03E+08	G	A	DYNC2H1	nonsynonymous SNV	4.20E-03
11	1.1E+08	1.1E+08	G	A	ZC3H12C	nonsynonymous SNV	2.50E-05
11	1.18E+08	1.18E+08	T	A	MPZL2	nonsynonymous SNV	0.0021
11	1.18E+08	1.18E+08	T	A	KMT2A	nonsynonymous SNV	9.89E-05
11	1.21E+08	1.21E+08	G	A	TBCEL	nonsynonymous SNV	0.00004146
11	1.29E+08	1.29E+08	C	T	ARHGAP32	nonsynonymous SNV	0.0012
11	1.3E+08	1.3E+08	C	T	ADAMTS8	nonsynonymous SNV	0.0044
12	7354399	7354399	G	C	PEX5	nonsynonymous SNV	0.0023
12	91445287	91445287	C	T	KERA	nonsynonymous SNV	0.000008316
12	1.02E+08	1.02E+08	C	T	CHPT1	nonsynonymous SNV	0.0018
12	1.23E+08	1.23E+08	T	G	PITPNM2	nonsynonymous SNV	.
12	1.24E+08	1.24E+08	C	T	ATP6V0A2	nonsynonymous SNV	0.0004
12	1.32E+08	1.32E+08	G	A	MMP17	nonsynonymous SNV	1.80E-03
13	46952078	46952078	T	C	KIAA0226L	nonsynonymous SNV	0.0045
13	96540168	96540168	G	A	UGGT2	nonsynonymous SNV	0.0008
13	1.03E+08	1.03E+08	T	C	CCDC168	nonsynonymous SNV	0.0035
14	71209128	71209128	C	T	MAP3K9	nonsynonymous SNV	1.20E-03
14	94845944	94845944	C	A	SERPINA1	nonsynonymous SNV	0.0023
14	1.01E+08	1.01E+08	C	G	RTL1	nonsynonymous SNV	.
14	1.03E+08	1.03E+08	C	T	TECPR2	nonsynonymous SNV	0.00001681
14	1.05E+08	1.05E+08	C	G	C14orf79	nonsynonymous SNV	0.0017
15	29561606	29561606	T	C	NDNL2	nonsynonymous SNV	0.0011
15	65116504	65116504	C	G	PIF1	nonsynonymous SNV	0.0026
15	78390805	78390805	C	T	SH2D7	nonsynonymous SNV	0.00003318
16	1273558	1273558	C	T	TPSG1	nonsynonymous SNV	0.002
16	2042912	2042912	A	G	SYNGR3	nonsynonymous SNV	.
16	30000929	30000929	A	G	TAOK2	nonsynonymous SNV	0.0002
17	8093324	8093324	C	G	C17orf59	nonsynonymous SNV	0.0036

17	17942915	17942915	A	C	GID4	nonsynonymous SNV	.
17	26691491	26691500	CAC-TGAGGTG	-	SEBOX	frameshift deletion	0.0009
17	34091112	34091112	G	A	C17orf50	nonsynonymous SNV	0.0002
17	37262135	37262135	C	A	PLXDC1	nonsynonymous SNV	.
17	37879585	37879585	A	G	ERBB2	nonsynonymous SNV	4.80E-03
17	38975259	38975259	C	T	KRT10	nonsynonymous SNV	1.60E-03
17	39115095	39115095	G	A	KRT39	nonsynonymous SNV	0.0002
17	40318398	40318398	C	T	KCNH4	nonsynonymous SNV	7.00E-04
17	66937004	66937004	G	A	ABCA8	nonsynonymous SNV	6.00E-04
17	72997464	72997464	G	A	CDR2L	nonsynonymous SNV	0.0002
17	74056418	74056418	T	C	SRP68	nonsynonymous SNV	0.0022
17	74382047	74382048	TT	-	SPHK1	frameshift deletion	0.0027
17	76046849	76046849	C	T	TNRC6C	nonsynonymous SNV	1.40E-03
17	79987456	79987456	G	A	LRRC45	nonsynonymous SNV	2.70E-03
17	80395194	80395194	G	A	HEXDC	nonsynonymous SNV	.
17	81052105	81052105	G	A	METRNL	nonsynonymous SNV	0.0005
18	48190630	48190630	C	T	MAPK4	nonsynonymous SNV	0.00004145
18	55365047	55365047	T	C	ATP8B1	nonsynonymous SNV	8.00E-04
18	60646017	60646017	C	G	PHLPP1	nonsynonymous SNV	0.0026
19	1468408	1468408	C	A	APC2	nonsynonymous SNV	0.0008
19	2210451	2210451	C	G	DOT1L	nonsynonymous SNV	3.20E-03
19	2934625	2934625	C	T	ZNF77	nonsynonymous SNV	0.0018
19	8587708	8587708	C	A	MYO1F	nonsynonymous SNV	4.00E-03
19	12800601	12800601	G	A	FBXW9	nonsynonymous SNV	0.0001
19	17534540	17534540	G	T	MVB12A	nonsynonymous SNV	0.0014
19	17945708	17945708	C	G	JAK3	nonsynonymous SNV	4.00E-04
19	19381028	19381028	C	T	TM6SF2	nonsynonymous SNV	3.00E-04
19	19612832	19612832	A	C	GATAD2A	nonsynonymous SNV	0.00005814
19	33465099	33465099	C	T	C19orf40	nonsynonymous SNV	5.00E-03
19	35760880	35760880	C	T	USF2	nonsynonymous SNV	0
19	36018115	36018115	C	T	SBSN	nonsynonymous SNV	0.0001
19	42753218	42753218	G	A	ERF	nonsynonymous SNV	0.0005
19	47910643	47910643	C	T	MEIS3	nonsynonymous SNV	0.0007
19	48305694	48305694	A	G	TPRX1	nonsynonymous SNV	0.00005312
19	50097746	50097746	G	A	PRR12	nonsynonymous SNV	0.0019
19	51329146	51329146	C	T	KLK15	nonsynonymous SNV	2.50E-03
19	51649313	51649313	G	A	SIGLEC7	nonsynonymous SNV	7.41E-05
19	51957493	51957493	T	C	SIGLEC8	nonsynonymous SNV	0.0032
19	51957497	51957497	C	G	SIGLEC8	nonsynonymous SNV	0.0026
19	52000192	52000192	A	G	SIGLEC12	nonsynonymous SNV	0.0028
19	54848826	54848826	C	T	LILRA4	nonsynonymous SNV	0.0038
19	59012675	59012675	C	T	SLC27A5	nonsynonymous SNV	0.0023
20	33508358	33508358	T	C	ACSS2	nonsynonymous SNV	0.0028
20	37632435	37632435	G	A	DHX35	nonsynonymous SNV	0.000008237
20	42694674	42694674	A	C	TOX2	nonsynonymous SNV	.
20	48552981	48552981	C	G	RNF114	nonsynonymous SNV	.
20	51802198	51802198	C	T	TSHZ2	nonsynonymous SNV	0.00008139
20	52774635	52774635	A	G	CYP24A1	nonsynonymous SNV	0.0007
21	16015325	16015325	C	T	LOC388813	nonsynonymous SNV	0.0036
21	34923159	34923159	A	G	SON	nonsynonymous SNV	.
22	21384032	21384032	G	A	SLC7A4	nonsynonymous SNV	0.00003434
22	25124155	25124155	C	T	PIWIL3	nonsynonymous SNV	0.0041

22	29445841	29445841	G	A	ZNRF3	nonsynonymous SNV	0.00007248
22	31032952	31032952	T	G	SLC35E4	nonsynonymous SNV	0.004
22	37964703	37964703	G	A	CDC42EP1	stopgain	0.00005552
22	38129388	38129388	G	A	TRIOBP	nonsynonymous SNV	0.0032
22	38512190	38512190	G	A	PLA2G6	nonsynonymous SNV	.
X	1.09E+08	1.09E+08	C	T	AMMECR1	nonsynonymous SNV	0.0001
X	1.24E+08	1.24E+08	-	A	LOC100129520	frameshift insertion	.-
